# Delta‐like ligand‐4 regulates Notch‐mediated maturation of second heart field progenitor‐derived pharyngeal arterial endothelial cells

**DOI:** 10.1111/jcmm.17542

**Published:** 2022-09-09

**Authors:** Prashan De Zoysa, Omar Toubat, Drayton C. Harvey, Christopher Yi, Jiang Liu, Susana Cavallero, Young‐Kwon Hong, Henry M. Sucov, Subramanyan Ram Kumar

**Affiliations:** ^1^ Department of Surgery, Keck School of Medicine University of Southern California Los Angeles California USA; ^2^ Division of Cardiology, David Geffen School of Medicine University of California Los Angeles Los Angeles California USA; ^3^ Department of Regenerative Medicine and Cell Biology Medical University of South Carolina Charleston South Carolina USA; ^4^ Department of Pediatrics, Keck School of Medicine University of Southern California Los Angeles California USA

**Keywords:** arch artery, delta‐like ligand‐4, Dll4, Notch signalling, PAA, second heart field, SHF

## Abstract

Mesodermal progenitors in the second heart field (SHF) express *Delta‐like‐ligand 4* (*Dll4*) that regulates Notch‐mediated proliferation. As cells of SHF lineage mature to assume endocardial and myocardial cell fates, we have shown that *Dll4* expression is lost, and the subsequent expression of another Notch ligand *Jagged1* regulates Notch‐mediated maturation events in the developing heart. A subset of SHF progenitors also matures to form the pharyngeal arch artery (PAA) endothelium. Dll4 was originally identified as an arterial endothelial‐specific Notch ligand that plays an important role in blood vessel maturation, but its role in aortic arch maturation has not been studied to date secondary to the early lethality observed in *Dll4* knockout mice. We show that, unlike in SHF‐derived endocardium and myocardium, *Dll4* expression persists in SHF‐derived arterial endothelial cells. Using SHF‐specific conditional deletion of *Dll4*, we demonstrate that as SHF cells transition from their progenitor state to an endothelial fate, Dll4‐mediated Notch signalling switches from providing proliferative to maturation cues. *Dll4* expression maintains arterial identity in the PAAs and plays a critical role in the maturation and re‐organization of the 4th pharyngeal arch artery, in particular. Haploinsufficiency of *Dll4* in SHF leads to highly penetrant aortic arch artery abnormalities, similar to those observed in the clinic, primarily resulting from aberrant reorganization of bilateral 4th pharyngeal arch arteries. Hence, we show that cells of SHF lineage that assume an arterial endothelial fate continue to express *Dll4* and the resulting Dll4‐mediated Notch signalling transitions from an early proliferative to a later maturation role during aortic arch development.

## 1 INTRODUCTION

Early during embryogenesis, blood exits the developing heart through an undivided outflow tract before being circulated dorsally and laterally through paired pharyngeal arch arteries (PAAs) into twin dorsal aortae. These dorsal aortae combine caudally to form a single aorta.^1^ Between embryonic day (E) 8.5 and E14.5, the PAAs and the dorsal aorta undergo complex rearrangements eventually forming the mature aortic arch phenotype seen in adults. While the first and second PAAs contribute to arteries that supply portions of the face, the third arch arteries give rise to the right and left carotid arteries. The left and right fourth PAAs contribute to the aortic arch and portions of the right subclavian artery, respectively.^1^ The right and left sixth PAAs contribute to the right and left pulmonary arteries. The left sixth PAA additionally contributes to the ductus arteriosus, as well. The right dorsal aorta gives rise to a portion of the right subclavian artery while the left dorsal aorta gives rise to the distal aortic arch and the descending aorta.^1^ Such an intricate reorganization process is tightly regulated by spatially and temporally varying molecular signals.

Delta‐like ligand‐4 (Dll4) was originally described as a unique arterial endothelial‐specific ligand of the Notch receptor.^2^ Haploinsufficiency of Dll4 results in an arterial maturation arrest and embryonic lethality by E9.5.^3^, ^4^, ^5^ The early lethality in global knockouts precludes evaluation of the role of Dll4 in arteries that mature later during development. This is particularly true for PAAs, which not only mature later in embryonic life, but, more importantly, are also formed by endothelial cells that are derived from second heart field (SHF) progenitor cells.^6^ SHF progenitors are splanchnic mesodermal cells that express cardiac‐specific markers and mature into endocardial, myocardial and endothelial cells.^7^ We have shown that *Dll4* is expressed from E8.5 by SHF cells and Dll4‐mediated Notch signalling plays a crucial role in SHF proliferation, thereby maintaining the pool of cells of SHF lineage required for development of the right ventricle (RV) and cardiac outflow tract (OFT).^8^ These studies also demonstrated that *Dll4* expression is lost in SHF‐derived cardiac structures by E12.5–14.5. However, the fate and biological role of Dll4 in SHF‐derived endothelial cells that form the aortic arch have not been formally evaluated.

To that end, we sought to evaluate the role of *Dll4* expression in PAA maturation and development of the aortic arch. Unlike in SHF‐derived cardiac structures, we demonstrate that *Dll4* expression persists in SHF‐derived PAA endothelial cells. Haploinsufficiency of *Dll4* in SHF leads to loss of arterial identity, due in part to loss of *Hey1* expression, and lack of maturation of nascent arteries. This results in highly penetrant aortic arch defects that mirror clinically relevant aortic arch abnormalities.

## 2 METHODS

### 2.1 Mice

All animal experiments were carried out under protocols approved by the Institutional Animal Care and Use Committee of the University of Southern California. *Islet1‐Cre* ^9^ and *Mef2c‐AHF‐Cre*,^10^ *Wnt1‐Cre*,^11^ *Rosa26‐tdTomato* (*R26RtdT*)^12^ and *CBF:H2B‐Venus Notch* Reporter^13^ mouse lines have all been previously described. The Cre gene was maintained on the paternal side to eliminate risk of germline transmission. *Dll4* ^*FF*^ mice were generated in Duarte Lab and previously reported.^3^, ^14^ *Dll4‐F2‐LacZ* mice have also been previously described.^15^ Embryo dissection was carried out by standard methods. Genotyping was undertaken using standard PCR techniques and specific primers used are listed in Table S1.

### 2.2 Tissue analysis and histology

Immunofluorescence (IF), in situ hybridization (ISH), X‐gal staining and Haematoxylin–Eosin stains were performed using standard techniques. The antibodies used for staining are listed in Table S2. Standard validation techniques included deletion of primary or secondary antibody, or use of blocking peptide to validate antibody specificity, as appropriate. The *Dll4* probe used for in situ hybridization has been previously described.^14^

### 2.3 India ink Injection

For India ink injections, embryos were dissected in cold PBS at E10.5 and E12.5. Chest wall and pericardial tissues were carefully dissected to expose the heart, and glass micropipettes were used to inject ink into the primitive ventricle and OFT. Embryos were incubated in 4% paraformaldehyde overnight at 4°C. Whole mount bright field imaging was performed, with frontal and right and left lateral images to evaluate arch artery organization.

### 2.4 Human umbilical arterial endothelial cells (HUAEC) tube formation assay

HUAEC were treated with control, *Dll4* or *Hey1* siRNA (50 nM) as appropriate. Dextran‐coated Cytodex 3 microcarriers were coated with HUAEC, resuspended in fibrinogen and added to wells of a 24‐well plate containing thrombin to allow clotting. 1 ml of endothelial basal media containing 2% foetal bovine serum (FBS) with or without recombinant VEGF (2.5 ng/ml) was added to each well, and dermal fibroblasts were plated on top of the clot. Beads were photographed at 5× magnification after 3–5 days in culture.

## 3 RESULTS

### 3.1 Pharyngeal arch arteries express Dll4 at appropriate time points during their re‐organization

**FIGURE 1:**
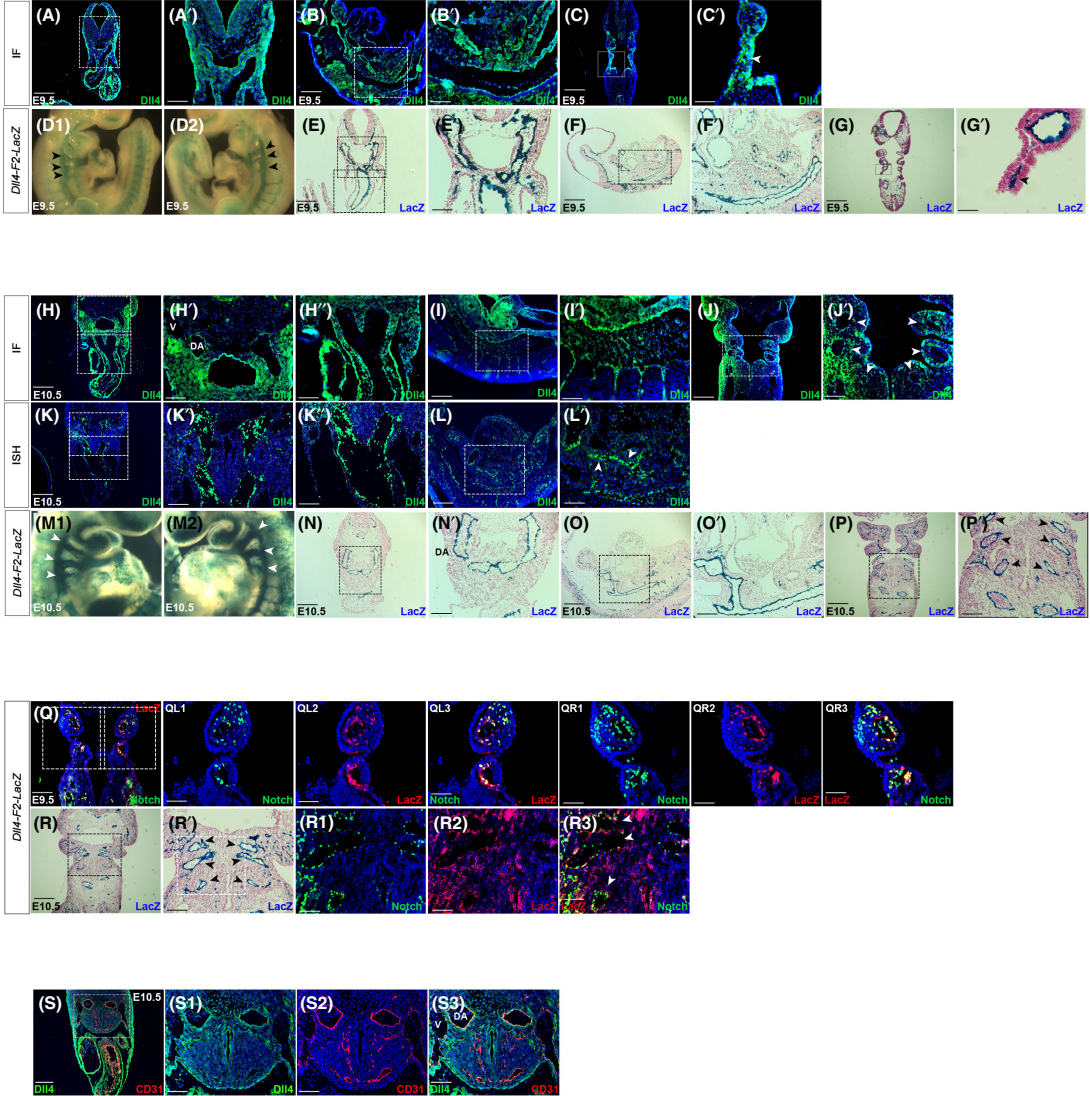
Dll4 is expressed by pharyngeal arch artery endothelium and the dorsal aorta. Representative images of E9.5 and E10.5 embryos are shown. Dll4 protein expression (immunofluorescence, IF) was studied in E9.5 transverse (A), sagittal (B) and coronal (C) fixed‐frozen sections. Dll4 is expressed in the cardiac OFT connecting to the 4th pharyngeal arch artery and then to the dorsal aorta (A and higher magnification of boxed area of A in A′). Dll4 is expressed in the pharyngeal mesodermal region as well as expected. Sagittal sections also show that Dll4 is expressed in the OFT as well as the dorsal aorta (B and higher magnification of boxed area of B in B′). Coronal sections show 3rd, 4th and 6th pharyngeal arch arteries expressing Dll4 (C and higher magnification of boxed area of C in C′). X‐gal staining in *Dll4‐F2‐LacZ* embryos was used as a complementary method to assess *Dll4* expression both in whole mount (D) and sections (E‐G). Whole mount lacZ staining reveals X‐gal signals in the 3rd, 4th and 6th pharyngeal arch arteries bilaterally (arrowheads in D1 and D2). Sections show X‐gal staining pattern similar to IF in transverse (E), sagittal (F) and coronal (G) sections. E10.5 embryos also demonstrate Dll4 expression on IF in the pharyngeal mesoderm, 4th pharyngeal arch artery and developing dorsal aorta (DA), and intersomitic vessels (H–J). *Dll4* transcript expression evaluated through in situ hybridization (ISH) (K, L) also showed similar expression pattern. Cardinal vein (V) adjacent to dorsal aorta (DA) (H′) does not express Dll4, as expected. X‐gal staining in *Dll4‐F2‐LacZ* embryos at E10.5 shows Dll4 expression in the bilateral 3rd, 4th and 6th pharyngeal arch arteries in whole mount examination (arrowheads in M1 and M2). Sections (N–P) confirm expression pattern seen with IF. Arrowheads in P′ represent the bilateral 3rd, 4th and 6th pharyngeal arch arteries. Coronal sections of *Dll4‐F2‐LacZ* and *CBF:H2B‐Venus Notch* Reporter embryos were then evaluated by LacZ immunostaining. At E9.5, LacZ positivity and Notch activity are seen in endothelial cells in both the left (Q, QL1–Ql3) and right (Q, QR1–QR3) 3rd and 4th pharyngeal arch arteries. This co‐localization was evident at E10.5 as well (R1–R3). Arrowheads in R′ and R3 represent the 3rd, 4th and 6th pharyngeal arch arteries. There is co‐localization of Dll4 and CD31 expression in (arterial) endothelial elements in the pharyngeal mesoderm and dorsal aorta (DA). Adjacent cardinal vein is CD31‐positive (S2), but Dll4‐negative (S1) confirming specificity of Dll4 signal. DA, Dorsal aorta; V, Vein. Wholemount magnification: ×3 (D1, D2, M1, M2). Scale Bars: 50 μm (C′, R1–R3), 75 μm (G′), 100 μm (A′, B′, H′, H″, I′, J′, K′, K″, L′, QL1–QL3, QR1–QR3, S1–S3), 150 μm (E′, F′, N′, O′, P′, R′), 250 μm (A–C, H, I–L, Q, S), 300 μm (E–G, N–P, R).

We studied Dll4 protein (IF) and transcript (ISH) expression in PAAs at relevant time points during their formation and re‐organization (Figure 1). As an additional technique, we used stable transgenic founder mouse lines in which the non‐coding region (F2) in the third intron of *Dll4* drives a minimum promoter LacZ reporter (*F2‐LacZ*).^16^ LacZ expression serves as a surrogate for *Dll4* expression in these animals. This enhancer element was identified specifically for activity in arterial endothelial elements, and as such, this model is particularly relevant to study Dll4 expression in developing arch arteries.

At E9.5, Dll4 expression is evident in transverse sections of the fourth PAA extending out from the OFT (Figure 1A,A′) and in the developing dorsal aorta in sagittal sections (Figure 1B,B′). Dll4 expression is also evident in the fourth PAA in coronal sections (Figure 1C,C′). Whole mount X‐gal staining in *F2‐LacZ* mice reveals LacZ signals indicating Dll4 expression in 3rd, 4th and 6th PAAs bilaterally (Figure 1D1,D2). Transverse, sagittal and coronal sections of these mice confirm Dll4 expression in the same areas as demonstrated by IF (Figure 1E–G′). Dll4 protein expression persists in bilateral 3rd, 4th and 6th PAAs, dorsal aorta and intersomitic vessels at E10.5 as well (Figure 1H–J′). In situ hybridization reveals *Dll4* mRNA expression in 4th PAA and dorsal aorta in transverse (Figure 1K,K′) and sagittal (Figure 1L,L′) sections. Similarly, whole mount X‐gal staining and sections in *F2‐LacZ* mice confirms Dll4 expression in 3rd, 4th and 6th PAAs bilaterally (Figure 1M–P′).

To evaluate the activity of Notch receptor in these cells, we crossed *F2‐LacZ* mice with a *CBF:H2B‐Venus Notch* reporter line, where Notch signalling results in nuclear expression of YFP. We studied LacZ expression in sections by IF and X‐gal staining. Endothelial cells in the 3rd, 4th and 6th PAAs that express Dll4 also demonstrate Notch activity (nuclear YFP) at E9.5 (Figure 1Q,QL1–QR3) and E10.5 (Figure 1R,R′,R1–R3), indicating that the Dll4 is biologically active. Finally, to confirm Dll4 expression in vascular elements, we stained E10.5 sections for the endothelial‐specific marker, CD31 and Dll4. We were able to confirm co‐expression of Dll4 and CD31 in dorsal aorta (DA in Figure 1‐S3), but lack of Dll4 expression in adjacent CD31‐positive cardinal vein (V in Figure 1‐S3), as anticipated.

### 3.2 *Dll4* expression in cells of SHF lineage is required for appropriate development of the aortic arch

**FIGURE 2:**
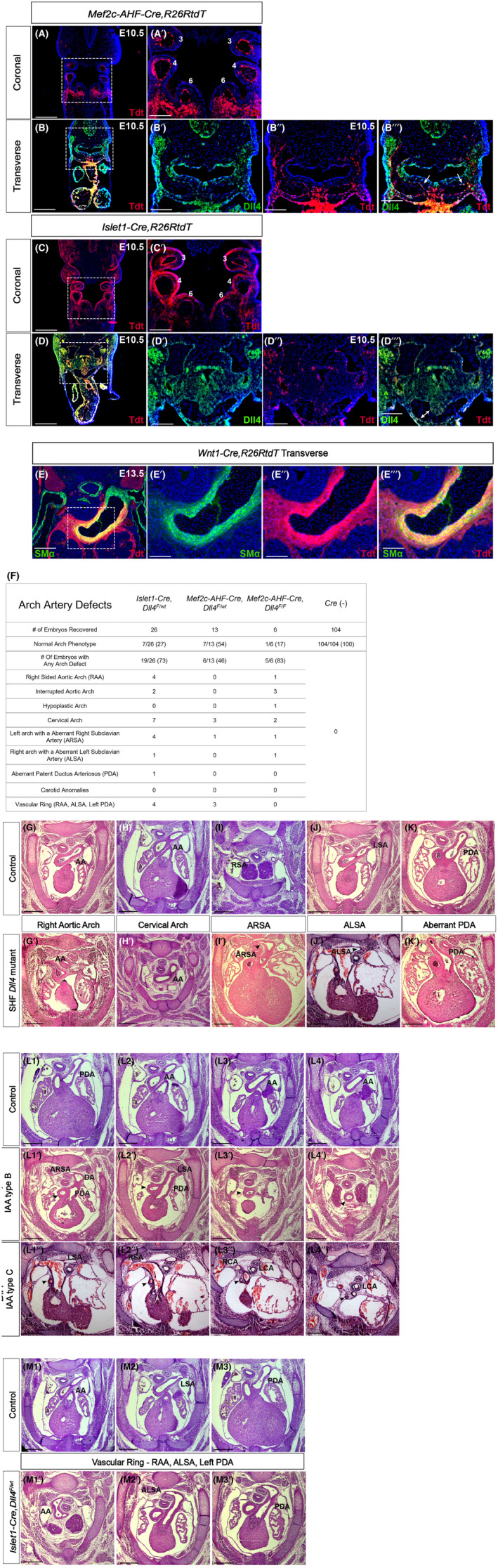
*Dll4* expression in SHF is required for appropriate development of the aortic arch. Coronal sections of *Mef2c‐AHF‐Cre,R26RtdT* (A) and *Islet1‐Cre,R26RtdT* (C) embryos at E10.5 demonstrates that 3rd, 4th and 6th pharyngeal arch artery endothelium is SHF‐derived. Similarly transverse sections of *Mef2c‐AHF‐Cre,R26RtdT* (B) and *Islet1‐Cre*, *R26RtdT* (D) co‐stained with Dll4 confirm lineage‐traced cells in 4th arch co‐express Dll4 (arrows in B‴ and D‴). Smooth muscle alpha actin stain in *Wnt1‐Cre,R26RtdT* embryos at E13.5 (E) demonstrates that the smooth muscle medial layer of the aortic arch is neural crest derived. Conditional loss of *Dll4* in SHF was achieved using *Islet1* and *Mef2c*‐mediated Cre recombinase expression. Haematoxylin and eosin‐stained transverse sections of E14.5 Cre‐negative littermate control embryos and SHF‐*Dll4* mutants (*Mef2c‐AHF‐Cre*, *Dll4* ^*F/F*^, *Mef2c‐AHF‐Cre*, *Dll4* ^*F/wt*^, *Islet1‐Cre*, *Dll4* ^*F/wt*^) show variety of arch artery defects (F). These include right aortic arch (AA, G′), cervical arch (H′), aberrant right subclavian artery (ARSA, arrowhead in I′), aberrant left subclavian artery (ALSA, arrowhead in J′) and aberrant ductus arteriosus (PDA, K′). Both Type B (L1′–L4′) and Type C (L1″–L4″) interrupted aortic arch (IAA) were observed (arrowheads in L1′–L4″ denotes the ascending aorta). A right aortic arch with aberrant left subclavian artery and a left‐sided ductus resulted in a complete vascular ring (M1′–M3′). AA, aortic arch; ARSA, aberrant right subclavian artery; ALSA, aberrant left subclavian artery; LCA, left carotid artery; LSA, left subclavian artery; PDA, patent ductus arteriosus; RCA, right carotid artery; RSA, right subclavian artery. Scale bar – 100 μm (A′–E″), 250 μm (A–E), 300 μm (G–K′, L1–L4″, M1–M3′).

The endothelial lining of the 3rd, 4th and 6th PAAs is derived from cells of SHF lineage. In order to lineage trace SHF cells, we used the *Cre* recombinase gene under the control of a *Islet1* promoter (Knockin/Knockout) which is more broadly expressed or *Mef2c* enhancer transgene whose expression is more restricted to the anterior SHF only. Coronal sections of *Mef2c‐AHF‐Cre*,*R262RtdT* (Figure 2A,A′) and *Islet1‐Cre,R262RtdT* (Figure 2C,C′) embryos at E10.5 confirm that PAA endothelial cells express tdT and are hence SHF‐derived.

To specifically demonstrate expression in cells of SHF lineage in the PAAs, we co‐stained transverse sections of both *Mef2c‐AHF‐Cre,R26RtdT* (Figure 2B–B‴) and *Islet1‐Cre,R26RtdT* (Figure 2D–D‴, Figure S1A–A‴) E10.5 embryos for Dll4. The 4th PAA clearly demonstrates co‐localization of tdT and Dll4 stains indicating that *Dll4* is expressed by cells of SHF lineage in the developing PAAs. We evaluated corresponding wildtype transverse sections for *Dll4* mRNA expression, which further confirmed *Dll4* transcripts in the 4th PAA (Figure S1B,B′). Lastly, comparable transverse sections from *F2‐LacZ* embryos also demonstrate X‐gal stain in the 4th PAA indicating *Dll4* expression (Figure S1C,C′). *Dll4* expression is also observed in cells of SHF lineage in the pharyngeal mesoderm as well as in the developing heart in these sections as we have previously shown.^8^ By E12.5, the PAA endothelium acquires a smooth muscle cell coat. Using neural crest‐specific marker, *Wnt1‐Cre,R26RtdT* in E13.5 embryos, we confirm that in our model the smooth muscle coat is derived from cardiac neural crest cells (Figure 2E–E‴).

To study the biological role of Dll4 in SHF progenitors that form the PAA endothelium, we employed SHF‐specific Cre‐mediated conditional knockout of *Dll4* expression using both *Islet1‐Cre* and *Mef2c‐AHF‐Cre* lines. Loss of Dll4 expression in PAAs was confirmed in conditional mutants by staining for Dll4 (Figure S2A–B′). Jagged1, the other Notch ligand of significance in heart development, is not expressed in the heart during this early time period as we have previously shown.^8^ We confirmed lack of expression of *Jagged1* in PAAs of control mice at E9.5 (Figure S2C1–C3) and lack of compensatory expression of *Jagged1* in PAAs of mutant embryos with SHF‐specific loss of *Dll4* (Figure S2C4–C6). We also confirmed that loss of *Dll4* in SHF did not result in change in expression of Notch receptors with relevance to vascular development, viz. *Notch1* (Figure S2D1–D6) and Notch4 (Figure S2E1–E6). Homozygous *Dll4* knockout in *Islet1‐Cre* background resulted in very early embryonic lethality as we have previously shown,^8^ precluding evaluation of PAA phenotypes. Similarly, only six (6% compared with expected 25%) *Mef2c‐AHF‐Cre,Dll4* ^*F/F*^ embryos could be recovered at E14.5 and 5/6 (83%) demonstrated arch artery defects (Figure 2F). However, embryos with haploinsufficiency (heterozygous loss) of *Dll4* in SHF were recovered at Mendelian numbers at E14.5 in both backgrounds and demonstrated highly penetrant arch artery defects (Figure 2F), allowing us to evaluate the mechanistic basis of these defects in heterozygous mutants.

**FIGURE 3:**
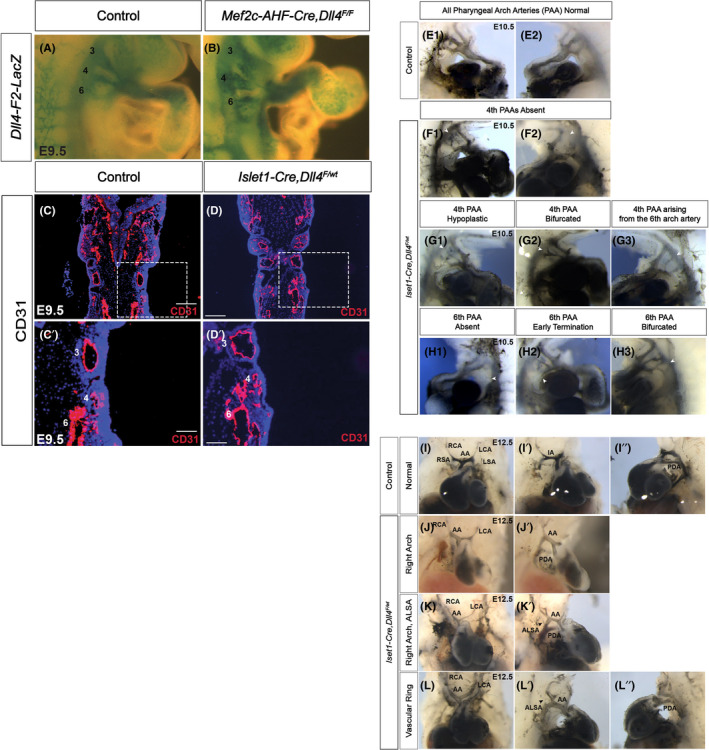
Haploinsufficiency of *Dll4* in SHF leads to defects in the re‐organization of pharyngeal arch arteries. Conditional loss of *Dll4* in SHF was achieved using *Islet1* and *Mef2c*‐mediated Cre recombinase expression. PAA anatomy was studied at E9.5 through whole mount analysis of *F2‐LacZ* mice in littermate control embryos (A) and *Mef2c‐Cre*, *Dll4* ^*F/F*^ mutants (B) showing normal PAA assembly. Similarly coronal sections of E9.5 control (C, C′) and *Islet1‐Cre,Dll4* ^*F/wt*^ mutants (D, D′) stained for CD31 show proper PAA early assembly in mutants. Whole mount examination at E10.5 of India ink injected littermate control embryos (E1, E2) and *Islet1‐Cre,Dll4* ^*F/wt*^ mutants demonstrate variety of PAA defects including complete loss (F1, F2), hypoplasia (G1), or inappropriate bifurcation (G2) of the 4th PAA, or origin of the 4th PAA from the 6th PAA (G3). Additionally, defects with the 6th PAA including complete loss (H1), early termination (H2) or bifurcation (H3) were also observed. Whole mount examination at E12.5 of India ink injected littermate control (I) and *Islet1‐Cre,Dll4* ^*F/wt*^ mutant embryos (J–L) demonstrate isolated right aortic arch (J, J′), right arch with aberrant left subclavian artery (K, K′) and complete vascular ring (L–L″). Arrowheads in K′ and L′ denotes the aberrant left subclavian arteries (ALSA). AA, aortic arch; ALSA, aberrant left subclavian artery; IA, innominate artery; LCA, left carotid artery; LSA, left subclavian arteryPDA, patent ductus arteriosus; RCA, right carotid artery; RSA, right subclavian artery. Wholemount magnification: ×4 (I–L″); ×3 (A1–H3). 50 μm (C′, D′), 100 μm (C, D).

Overall, loss of *Dll4* led to a variety of arch artery defects (Figure 2F). The majority of embryos revealed 4th PAA‐related defects. These include a cervical aortic arch, wherein the aortic arch is derived from the 3rd PAA due to inappropriate resorption of the 4th PAA. The aortic arch is observed more cervically in these embryos around the region of the thymus gland as opposed to more caudally by the base of the atrial appendages in controls (Figure 2H′). Another common anomaly was aberrant right subclavian artery, wherein this artery arose from the distal arch/proximal descending aorta and travelled posterior to the trachea and oesophagus instead of from the innominate artery, which is the first branch of the aortic arch (Figure 3I′). This phenotype results from inappropriate resorption of the right 4th PAA. Less frequently, the mirror image phenotype of an aberrant left subclavian artery was seen in embryos with right‐sided aortic arch (Figure 2J′) due to inappropriate resorption of the left 4th PAA. Interrupted aortic arch (IAA) was seen in 11% of the embryos indicating loss of 4th PAA. We observed both IAA type B (aortic arch disruption between the left carotid and left subclavian arteries) (Figure 3L1′–L4′) and IAA type C (aortic arch disruption between the innominate and left carotid artery) (Figure 3L1″–L4″). Interestingly, 4 of these 5 mice also had aberrant right or left subclavian arteries indicating that both left and right 4th PAAs were inappropriately resorbed in these embryos. In 8 (18%) embryos, an aberrant ductus arteriosus (PDA) was seen defined as a PDA on the opposite side of the aortic arch (Figure 2K′). This represents a defect in maturation of the 6th PAA. Seven (16%) embryos demonstrated a right‐sided aortic arch with aberrant left subclavian artery (loss of left 4th PAA) along with a left‐sided PDA (defect in 6th PAA) leading to the clinically well‐recognized complete vascular ring phenotype (Figure 2M1′–M3′). Interestingly, none of the mutant embryos demonstrated any carotid artery abnormalities, implying normal 3rd PAA maturation. An isolated right‐sided aortic arch (Figure 2G′) was seen in 5 of 45 (11%) total mutant embryos, indicating that perturbation of PAA re‐organization impacts eventual resorption of right dorsal aortic arch in a small subset of embryos.

### 3.3 Dll4 regulates subsequent maturation of nascent PAAs

In order to evaluate the stage at which PAA development was perturbed, we first began by evaluating PAAs at E9.5. We studied PAA anatomy by whole mount analysis of *F2‐LacZ* mice (where LacZ expression defines arterial endothelium, Figure 3A,B) and by staining coronal sections for CD31 (Figure 3C,D). By both modalities, all control and mutant embryos evaluated showed normal, stage‐appropriate PAA patterning. This indicates that loss of Dll4 does not impact early PAA assembly. We then studied E10.5 embryos by India ink injection followed by whole mount evaluation. Conditional SHF‐*Dll4* mutant embryos begin to demonstrate a spectrum of 4th and 6th PAA defects by this stage. These defects manifest as complete loss (Figure 3F1,F2), hypoplasia (Figure 3G1), or inappropriate bifurcation (Figure 3G2) of the 4th PAA or improper origin of the 4th PAA from the 6th PAA (Figure 3G3). A smaller proportion of embryos also demonstrated 6th PAA defects. There was complete loss (Figure 3H1), early termination (Figure 3H2) or bifurcation (Figure 3H3) of 6th PAA. No 3rd PAA abnormality was observed. PAA re‐organization is well underway by E12.5. India ink evaluation of mutant embryos at this stage demonstrates eventual histologic phenotypes. We were able to demonstrate right aortic arch (Figure 3J,J′), right arch with aberrant left subclavian artery (Figure 3K,K′) and vascular ring (Figure 3L–L″) phenotypes.

**FIGURE 4:**
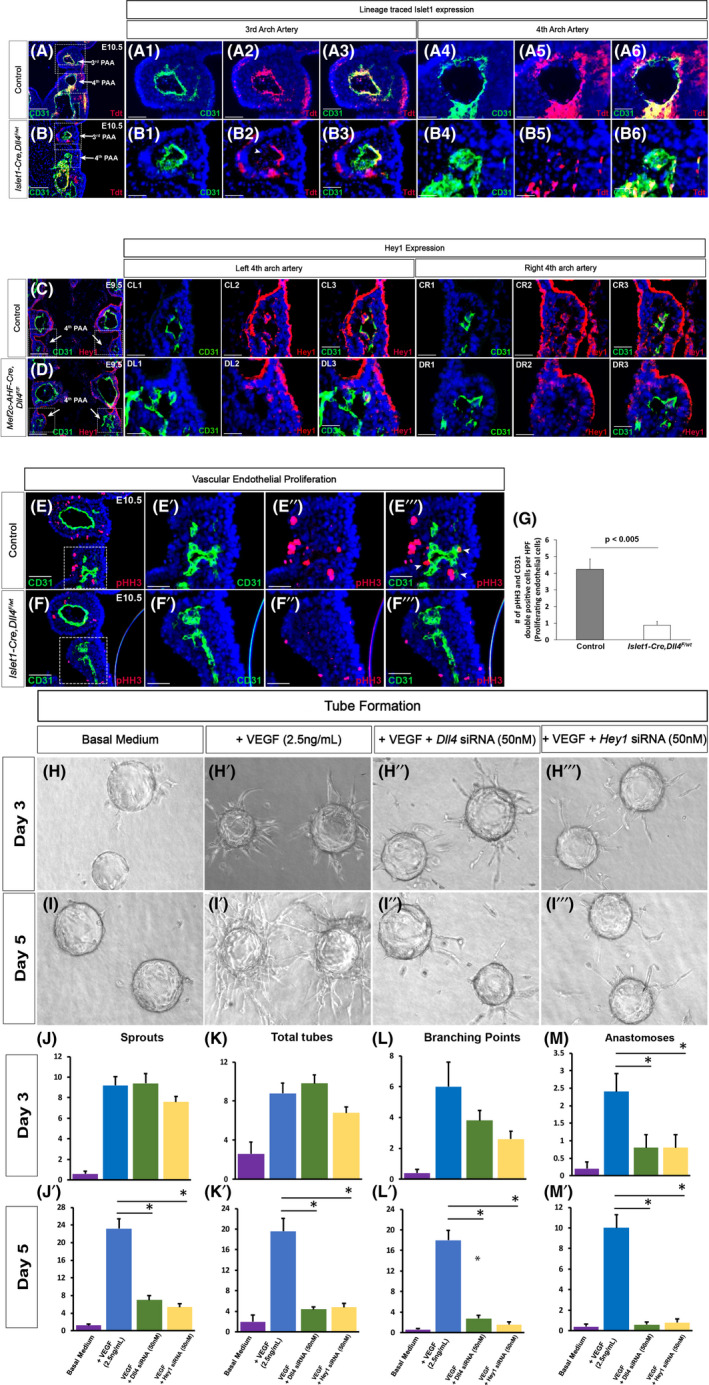
Dll4‐mediated *Hey1* expression regulates maturation of pharyngeal arch arteries. SHF cells were lineage traced by crossing *R26RtdT* mice into *Islet1‐Cre* line. Coronal sections of littermate control (A) and *Dll4* heterozygous mutant (*Islet1‐Cre,Dll4* ^*F/wt*^) (B) embryos were evaluated at E10.5. Mutant embryos maintain the calibre of the 3rd pharyngeal arch artery (B1 vs. A1) although less than half the artery is composed of SHF‐derived red cells compared with the entire artery in controls (B2 vs. A2). Arrowhead in B2 indicate the tdTomato negative non‐SHF‐derived arterial endothelium in the 3rd pharyngeal arch artery. In contrast, the 4th pharyngeal arch artery lacks lumen and is very small in mutants (B4 vs. A4), and all remaining cells are of SHF lineage as in controls (B5 vs. A5). Hey1 expression was evaluated in the 4th pharyngeal arch arteries in littermate control and *Mef2c‐AHF‐Cre,Dll4* ^*F/F*^ mutants. Hey1 expression is largely absent in both left (DL1‐3) and right (DR1‐3) sides of the mutants compared with the controls. Coronal sections of E10.5 control (E) and *Islet1‐Cre,Dll4* ^*F/wt*^ mutant (F) embryos were stained for pHH3 and CD31 to study endothelial cell proliferation. Higher magnification of boxed area in E and F demonstrate reduced proliferation in mutants (F′–F‴) compared with controls (E'–E‴). Arrowheads in E‴ denote pHH3 and CD31 double‐positive proliferating endothelial cells. CD31 and pHH3 double‐positive cells were counted in 6 control and 12 mutant fields (G, Mean, SEM) demonstrating an 80% reduction in proliferating SHF cells in mutants (*p* < 0.005). In vitro analysis of tube formation was performed using Human Umbilical Arterial Endothelial Cells (HUAEC) in fibrin gels. Representative pictures at 3 (H–H‴) and 5 (I–I‴) days are shown. Tube formation was studied under basal condition (H, I), with addition of VEGF (2.5 ng/mL, H′, I′) and using HUAEC pre‐treated with *Dll4* (H″, I″) or *Hey1* specific (H‴, I‴) siRNA (50 nM). (J–M′) Quantitation of total number of sprouts, tubes, branching points, and anastomosis in the fibrin bead assay averaged over 5 random high‐power fields in triplicate wells for each condition. The data are normalized to number of beads per field. Data are represented as mean ± SEM, *p < 0.05 versus VEGF alone. Wholemount magnification: ×5 (H–I‴). Scale Bars: 25 μm (A1–A6, B1–B6, CL1–CL3, CR1–CR3, DL1–DL3, DR1–DR3, E′‐F″), 50 μm (C, D, E, F), 100 μm (A, B).

Our analyses at E10.5 and E14.5 demonstrated no 3rd PAA defects. This would imply that 3rd PAA endothelium is able to overcome Dll4 loss in SHF. To study this in greater detail, we stained coronal sections of control and mutant lineage‐traced E10.5 embryos with CD31 (Figure 4A–B6). Mutant embryos demonstrated normal‐sized 3rd PAA compared to controls (Figure 4B1‐3 compared to A1‐3). Interestingly, whereas the entire 3rd PAA was tdT‐positive in control embryos (Figure 4A2) indicating that it was fully derived from cells of SHF lineage, less than half the vessel was tdT‐positive (Figure 4B2) in all mutant sections evaluated. This indicates that when there is loss of SHF‐derived endothelial Dll4, the 3rd PAA is able to compensate by incorporating endothelial cells derived from non‐SHF source, thereby maintaining an appropriate calibre 3rd PAA. In contrast, 3/4th of mutant embryos showed near complete loss of lumen of 4th PAA (Figure 1B4‐6), with a much smaller CD31‐positive vessel and no additional contribution by non‐SHF‐derived, tdT‐negative cells. The 4th PAA, therefore, is primarily reliant on SHF‐derived cells and is unable to compensate for loss of these cells to maintain its calibre. This would explain the preponderance of 4th PAA defects observed in mutant embryos. There was variable loss of 6th PAA calibre in mutant embryos with about 15% of them showing significant reduction in diameter and no compensation by non‐SHF cells, similar to 4th PAA.

### 3.4 Dll4 mediates arterial maturation via expression of *Hey1*

Hey1 is a known downstream target of Notch signalling. Previous reports of *Hey1* knockout embryos have shown defective development of the 4th PAA, similar to the phenotype we observe in our mutants.^16^ We, therefore, evaluated Hey1 expression in PAAs in our mutants. By E9.5, although PAAs are assembled with appropriate CD31 labelled cells, SHF‐*Dll4* mutant embryos demonstrate near complete loss of *Hey1* expression in the 4th PAA endothelium both on the left (Figure 4DL1–DL3) and right (Figure 4DR1–DR3) sides. Thus, these nascent vessels lack expression of Dll4‐mediated Notch downstream proteins at this stage. At E10.5, control 4th PAA endothelial cells continued to proliferate as demonstrated by pHH3 and CD31 double‐positive cells (Figure 4E–E‴). In contrast, mutant 4th PAA had no lumen and an 80% reduction in proliferating endothelial cells (Figure 4F–F‴,G), suggesting that these arteries sustained a growth arrest. We interpret these findings to indicate that the growth‐arrested nascent vessels are headed towards resorption resulting eventually in complete loss or small calibre of the aortic arch.

We then modelled vascular plexus formation and maturation using tube formation of HUAEC in fibrin gels.^17^ Using this assay, we measured initial endothelial cell sprouting at 3 days as a marker of early vascular assembly. We then studied the complexity of lumenized tubes in terms of branching and anastomosis at 5 days as a marker of maturation of nascent vasculature. In the absence of added factors, there was no significant vessel sprouting from HUAEC‐coated beads (Figure 4H–J). VEGF (2.5 ng/mL) induced robust vessel sprouting at 3 days (Figure 4H′,J) and formation of complex branching and anastomoses by 5 days (Figure 4I′,J′–M′) as previously reported.^18^ When HUAEC were pre‐treated with *Dll4* or *Hey1*‐specific siRNA and then coated on to the beads, early vessel sprouting appeared to be comparable in the presence of VEGF (Figure 4H″,H'″,J), replicating the in vivo finding that loss of Dll4 does not impact early vascular assembly. However, these vessels failed to continue to sprout or demonstrate any branching or anastomosis by day 5 (Figure 4I″,I‴,J′–M′). Such a maturation arrest led to stagnation or resorption of these nascent vessels. Thus, our in vitro results recapitulate our in vivo findings and confirm that Dll4 and Hey1 expression in arterial endothelial cells does not impact early vasculogenesis but rather promotes subsequent maturation of the nascent vascular plexus leading to branching and anastomosis.

### 3.5 *Dll4* expression in PAAs is required for arterial specification and development of smooth muscle coat

**FIGURE 5:**
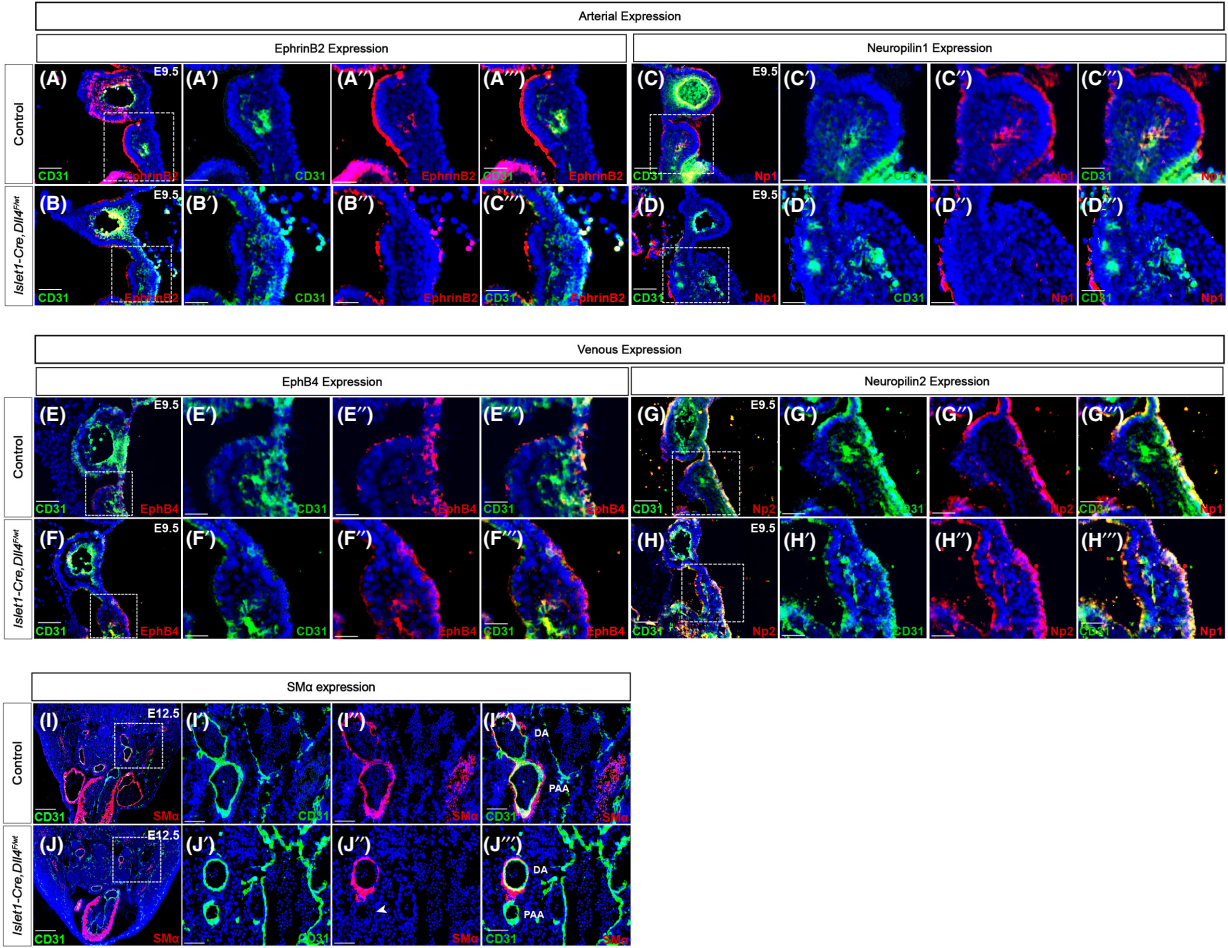
Dll4 expression in SHF is required for arterial identity in pharyngeal arch arteries. Expression of arterial markers EphrinB2 (A, B) and Neuropilin1 (C, D) in control and *Islet1‐Cre,Dll4* ^*F/wt*^ mutants demonstrate the lack of expression of EphrinB2 and Neuropilin1 in the 4th PAA of mutants (B″ and D″) compared with the controls (A" and C″). Similarly expression of venous markers EphB4 (E, F) and Neuropilin2 (G, H) in the 4th PAA of controls and *Islet1‐Cre,Dll4* ^*F/wt*^ mutants indicates the aberrant expression of these venous markers in the mutants (F″ and H″) compared with controls (E" and G"). Transverse sections at the level of distal aortic arch where the SHF‐derived 4th pharyngeal arch artery (PAAs in I‴ and J‴) meets the non‐SHF‐derived dorsal aorta (DA in I‴ and J‴) in control (I) and *Islet1‐Cre,Dll4* ^*F/wt*^ mutants (J) was stained for CD31 and smooth muscle alpha actin. Smooth muscle coverage is observed only around non‐SHF‐derived dorsal aorta in mutants compared with both arteries in control (J" vs. I″). Arrowhead in J" denotes the lack of expression of smooth muscle coat in SHF‐derived 4th pharyngeal arch artery. DA, Dorsal aorta; PAA, Pharyngeal arch artery. Scale Bars: 25 μm (A′–H″), 50 μm (A–H), 100 (I′–J‴), 250 μm (I, J).

*Dll4* is specifically expressed on arterial endothelial cells and its expression is an early event in arterial identity.^16^ We therefore evaluated the expression of arterial and venous markers in the 4th PAA of control and SHF Dll4 mutants (*Mef2c‐AHF‐Cre*, *Dll4* ^*F/F*^). By E9.5, mutant embryos demonstrated lack of expression of arterial markers EphrinB2 (Figure 5A″ vs. B″) and Neuropilin1 (Figure 5C″ vs. D″) in 4th PAA compared with controls. Consistent with the ability of 3rd PAA endothelium to overcome loss of *Dll4*, 3rd PAA in mutants demonstrates normal EphrinB2 and Neuropilin1 expression (Figure 3B,D). It has been shown that in the absence of induction of arterial markers, vessels express default venous markers. In SHF‐*Dll4* mutants, loss of arterial marker expression in 4th PAA was associated with aberrant expression of venous markers such as EphB4 (Figure 5E″ vs. F″) and Neuropilin2 (Figure 5G″ vs. H″). Thus, *Dll4* expression in cells of SHF lineage is required for SHF‐derived 4th PAA endothelial cells to express arterial identity during maturation.

A hallmark of arterial maturation during development is acquiring a smooth muscle cell covering over endothelial cell layer. Control embryos at E12.5 show normal calibre 4th PAA joining the dorsal aortic arch (Figure 5I). Both the 4th PAA (derived from SHF progenitors) and the dorsal aorta (at this level derived from non‐SHF mesoderm) have recruited SMα‐positive Smooth Muscle cells (Figure 5I″) indicating normal arterial maturation. In contrast, the 4th PAA is small in calibre with a normal dorsal aorta in mutant embryos (Figure 5J). Because *Dll4* expression is only lost in cells of SHF lineage in these mutants, only SHF‐derived 4th PAA lacks a smooth muscle coat whereas the non‐SHF‐derived dorsal aorta has acquired a normal smooth muscle coat (Figure 5J″). Taken together, our data would indicate that Dll4 expression in SHF mesodermal progenitors is required for arterialization of SHF‐derived endothelial elements in PAAs and for them to obtain a smooth muscle layer as part of the normal arterial maturation process.

## 4 DISCUSSION

Notch signalling has been shown to play an important role in development, in general, and heart development, in particular. In cells of SHF lineage, we have shown Dll4‐mediated Notch signalling maintains a proliferative phenotype to ensure that an adequate pool of progenitor cells is available for incorporation into the developing heart.^8^ As the SHF cells incorporated into the right ventricle and OFT assume endocardial and myocardial cell fates, *Dll4* expression wanes and Jagged1 expression begins to predominate. Jagged1‐mediated Notch signalling then controls maturation events such as ventricular compaction in cardiomyocytes^19^ and endocardial‐to‐mesenchymal transformation in endocardial cells that give rise to semilunar valves.^20^ In contrast, in the subset of SHF cells that assume an endothelial cell phenotype, *Dll4* expression persists.^8^ In this study, our results support a model in which, persistent expression of *Dll4* in SHF‐derived arterial endothelial cells, and the resulting Dll4‐mediated Notch signalling, is crucially required for maturation events that govern re‐organization of PAAs into post‐natal aortic arch phenotypes. As in other SHF‐derived cell types, Notch signalling transitions from a proliferation to maturation role in SHF‐derived PAA endothelial cells, as well. However, this transition is not associated with a switch in its primary ligand as observed in the heart such that, in arterial endothelial cells, the same ligand‐receptor interaction also directs arterial maturation. We speculate that the concomitant expression of other endothelial cell‐specific molecules facilitates such a unique behaviour.^16^

During gastrulation, angioblasts are directly specified from lateral plate mesodermal progenitors. Arteriovenous specification of the angioblasts is genetically determined. Dll4 is a unique, arterial‐specific Notch ligand and is one of the earliest markers of arterial identity.^2^ Its cognate receptors, Notch1 and Notch4, are subsequently expressed in the arterial endothelium, and Dll4‐mediated Notch signalling turns on the molecular programme that maintains arterial identity and facilitates maturation of the arterial vasculature. During this maturation process, nascent arterial endothelial cells interact with smooth muscle cells that are also derived from mesodermal progenitors. The biological role of Dll4 in arterial precursor cells directly derived from the mesoderm has been established.^3^, ^4^ Haploinsufficiency of *Dll4* leads to arteriovenous specification defects and a vascular maturation arrest resulting in early embryonic lethality. Contrarily, overexpression of *Dll4* expands arterialization of endothelial cells, which also results in failure of appropriate arteriovenous specification and embryonic lethality.^21^ Dll4 also plays an important role in the maturation of arteries at sites of neoangiogenesis in the adult under physiologic and pathologic conditions.^22^ The ascending aorta, proximal aortic arch and its branches represent a unique vascular bed during development. Although the endothelial cells that form these vessels are also mesodermal in origin, they do not undergo direct angioblast specification. In contrast, SHF mesodermal progenitors initially establish a cardiogenic molecular signature with the expression of molecules such as Islet1, Mef2c and Hand2. A subset of these SHF cells subsequently assumes an endothelial fate and begins to express endothelial‐specific molecular markers.

Our current study demonstrates that Dll4 maintains its role as an arterial maturation molecule as mesodermal cells pass through the various molecular signatures to form PAAs. We utilize nuclear YFP signal in CBF:H2B‐Venus Notch reporter line as a surrogate for Notch signalling to demonstrate that membrane‐expressed Dll4 is biologically active in this context. We also demonstrate the physiological effect of loss of Dll4 resulting in loss of expression of Notch target protein Hey1 in endothelial cells and functional consequences in vessel development, as would be predicted from in vitro models. This would suggest that Dll4 expressed on endothelial cells interacts with Notch receptors also expressed by these cells. Whether Dll4 also interacts with SHF‐derived non‐endothelial mesenchyme in the pharyngeal arch cannot be separately ascertained with available molecular tools. Dll4‐Notch signalling is generally thought to occur in trans between Dll4 expressed on one endothelial cell and Notch expressed on a neighbouring cell. While that is likely also true in the SHF, our data are further unable to verify whether signalling occurs in cis or trans. Regardless, Dll4‐mediated Notch signalling in PAA endothelium regulates *Hey1* expression, induces arterial identity and supports continued growth and maturation of nascent blood vessels. A second unique aspect of PAA endothelium is that these cells interact with smooth muscle cells that are ectodermal in origin, derived from cardiac neural crest cells. Our results indicate that PAA maturation is impacted earlier in development to smooth muscle specification of the neural crest cells in the pharyngeal arches. Prior studies have shown that *Notch* knockout in the cardiac neural crest primarily results in defects in ductus arteriosus with <10% embryos demonstrating defects in aortic arch phenotypes.^23^ Taken together with our results, we propose that endothelial Dll4 signals primarily into endothelial Notch rather than neural crest‐expressed Notch in pharyngeal arches. The arch artery phenotypes in mutant mice closely resemble the phenotypes observed in *transforming growth factor* (*TGF*‐beta) mutants. TGF‐beta subtypes have been demonstrated to play a crucial role in heart and arch artery development.^24^ In addition, there is also evidence to suggest that Smad signalling mediates crosstalk between Notch and TGF‐beta pathways.^25^, ^26^ Future studies are required to evaluate the impact of these interactions in greater detail.

Interestingly, the impact of loss of *Dll4* is most pronounced in the 4th PAA, with less penetrant involvement of 6th PAA and complete lack of involvement of 3rd PAA. A spectrum of 4th PAA defects is observed in mutant embryos, likely related to biological variability in the timing and extent of Cre‐mediated *Dll4* loss. In our model, there is variable involvement of the 6th PAA. Aberrant maturation of the 6th PAA manifests exclusively as ductus arteriosus defects with no discernible defect in pulmonary artery development. We speculate that the lack of pulmonary artery defects relates in part to the different cellular interactions that regulate pulmonary artery maturation compared with aortic maturation.^27^ The 3rd PAA, in contrast, is able to compensate for loss of *Dll4* in SHF by incorporating non‐SHF‐derived endothelial cells to maintain growth and maturation. It is conceivable that the 3rd PAA, in part related to its location, has greater access to endothelial cells derived directly from mesodermal progenitors, without passing through the cardiogenic molecular signature. Alternatively, the 3rd PAA may routinely harbour a small population of non‐SHF‐derived endothelial cells that are capable of proliferating and compensating for the loss of SHF‐derived endothelial cells, such as seen in the case of *Dll4* knockout. Future studies focused on dissecting these individual mechanisms are required to fully explain the lack of 3rd arch phenotypes in mutant mice.

Like other vascular beds,^14^ there is a dosage‐sensitive requirement of *Dll4* in SHF‐derived arterial endothelial cells. Almost 2/3rd of the embryos with heterozygous loss of *Dll4* in SHF demonstrate arch artery defects. In contrast, we have shown that only about 10% of heterozygous SHF *Dll4* mutants develop intra‐cardiac defects, such as double outlet right ventricle,^28^ indicating a more crucial role for *Dll4* in arterial development. *Islet1*‐mediated loss of *Dll4* had a more penetrant phenotype compared with *Mef2c*‐driven loss, likely because of the more global expression of *Islet1* in SHF. Although *Islet1‐Cre* also labels a subset of neural crest cells, we have shown that *Dll4* is not expressed in the neural crest.^28^ The arch artery defects seen in our mutants are highly reminiscent of clinical arch defects. Recent evidence has shown that Dll4^29^, ^30^ and Notch pathway^31^, ^32^, ^33^, ^34^ mutations are relevant in clinical congenital heart defects. It would be of merit to study the prevalence of *Dll4/Notch* mutations in children with isolated arch artery defects.

## 5 CONCLUSION

In summary, our data provide a novel paradigm in which cells of SHF lineage maintain Dll4 expression as they mature into endothelial cells. Dll4‐mediated Notch signalling switches from its proliferative role in early progenitors to a maturation role in arterial elements. Dll4 expression induces arterial identity in PAAs and regulates events that coordinate PAA re‐organization into the post‐natal aortic arch phenotype.

## AUTHOR CONTRIBUTIONS

**Prashan De Zoysa:** Conceptualization (equal); data curation (lead); formal analysis (equal); investigation (lead); methodology (lead); validation (equal); visualization (equal); writing – original draft (lead); writing – review and editing (lead). **Omar Toubat:** Data curation (equal); methodology (equal); validation (equal); writing – original draft (equal); writing – review and editing (equal). **Drayton Harvey:** Data curation (equal); methodology (equal); writing – original draft (equal); writing – review and editing (equal). **Christopher Yi:** Data curation (equal); methodology (equal). **Jiang Liu:** Data curation (equal); methodology (equal). **Susana Cavallero:** Resources (equal); validation (equal). **Young Kwon Hong:** Resources (equal); validation (equal). **Henry M Sucov:** Conceptualization (equal); resources (equal); validation (equal); visualization (equal). **Subramanyan Ram Kumar:** Conceptualization (equal); formal analysis (lead); funding acquisition (lead); project administration (lead); resources (equal); validation (equal); visualization (equal); writing – review and editing (equal).

## CONFLICT OF INTEREST

No competing interests were declared.

## Supporting information


Figure S1

Figure S2

Table S1

Table S2

## Data Availability

The data that support the findings of this study are available from the corresponding author upon reasonable request.
