# Supplementary figures and images for: Delta‐like ligand‐4 regulates Notch‐mediated maturation of second heart field progenitor‐derived pharyngeal arterial endothelial cells
Source: J Cell Mol Med. 2022 Sep 9;26(20):5181–94. doi: 10.1111/jcmm.17542 (PMC9575135; doi:10.1111/jcmm.17542)

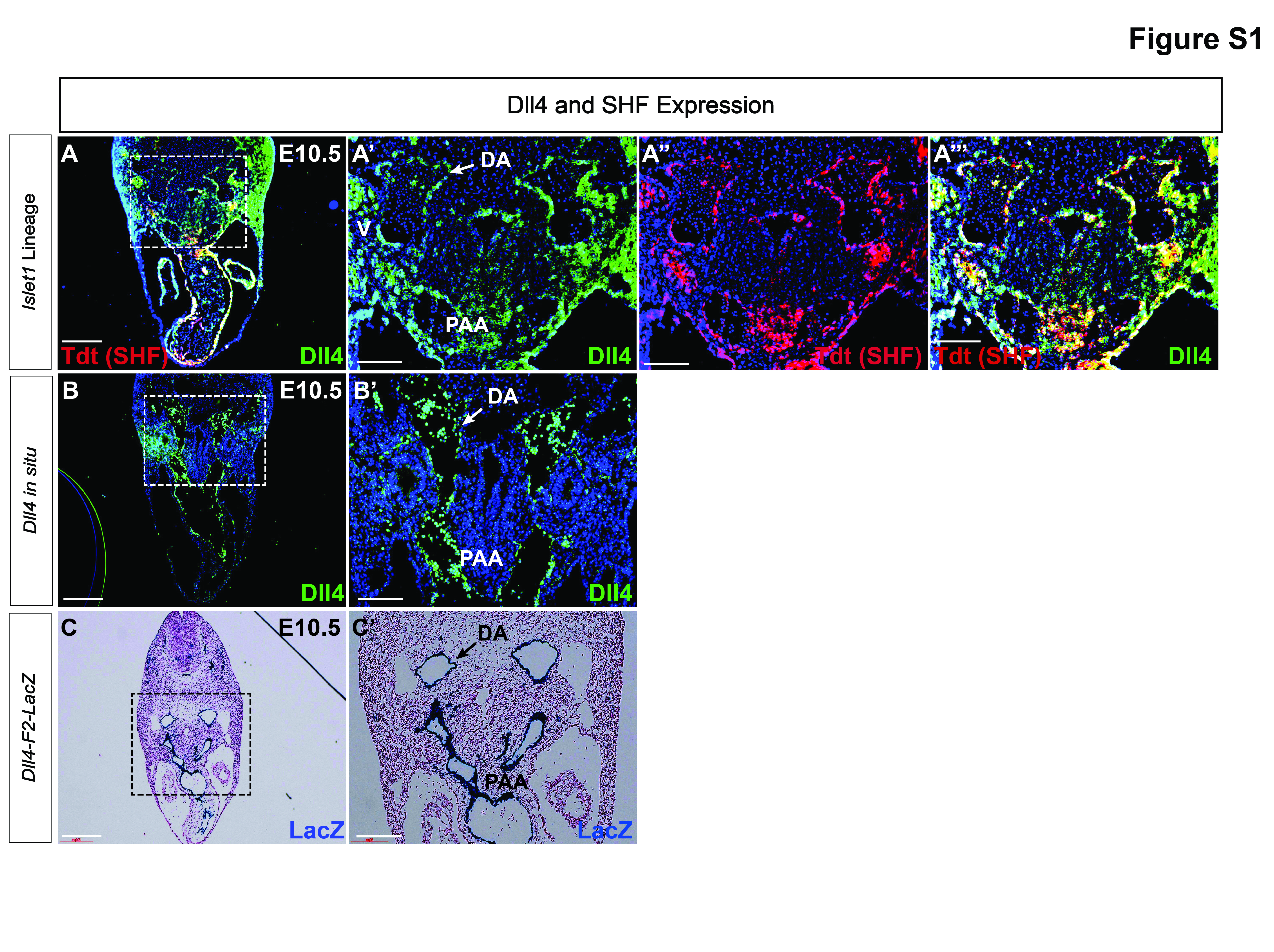

Supplement: Supplementary file 1 — Figure S1 [file JCMM-26-5181-s003.jpg]

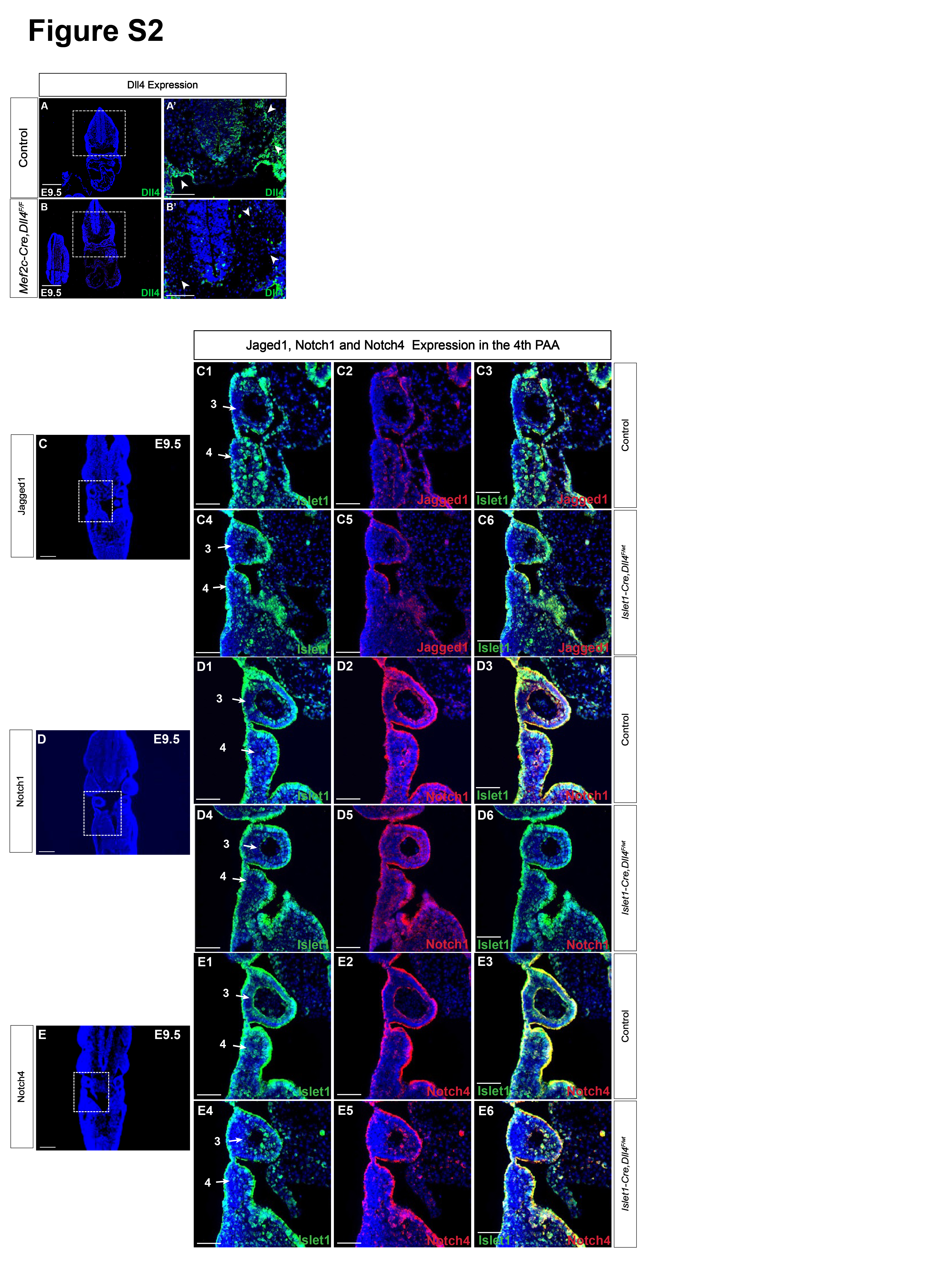

Supplement: Supplementary file 2 — Figure S2 [file JCMM-26-5181-s002.jpg]
