# Supplementary material for: Delta‐like ligand‐4 regulates Notch‐mediated maturation of second heart field progenitor‐derived pharyngeal arterial endothelial cells
Source: J Cell Mol Med. 2022 Sep 9;26(20):5181–94. doi: 10.1111/jcmm.17542 (PMC9575135; doi:10.1111/jcmm.17542)
Supplement: Supplementary file 3 — Table S1 [file JCMM-26-5181-s004.pdf]

Table S1

Primer	Sequence
**Dll4FF**	
Dll4-F	GTGCTGGGACTGTAGCCACT
Dll4-R	TGTTAGGGATGTCGCTCTCC
**Dll4-F2-LacZ**	
LacZ-F	ATCCTCTGCATGGTCAGGTC
LacZ-R	CGTGGCCTGATTCATTCC
**Mef2c-Cre**	
Mef2cCre-F	GAGCGTACGTGCTGCTTAGA
Mef2cCre-R	AATCGCGAACATCTTCAGGT
**Islet1-Cre**	
Islet1Cre Common-F	GCCACTATTTGCCACCTAGC
Islet1Cre Mutant allele-R	AGGCAAATTTTGGTGTACGG
Isl1Cre Wildtype allele-R	CAAATCCAAAGAGCCCTGTC
**Notch Reporter**	
Notch Forward	ACGTAAACGGCCACAAGTTC
Notch Reverse	AAGTCGTGCTGCTTCATGTG
**R26RtdTomato**	
Common Forward	AAGGGAGCTGCAGTGGAGTA
Wildtype Reverse	CCGAAAATCTGTGGGAAGTC
Mutant Reverse	CGGGCCATTTACCGTAAGTTAT
**Wnt1-Cre**	
Wnt1-Cre-F	CCTCTATCGAACAAGCATGCG
Wnt1-Cre-R	GCCAATCTATCTGTGACGGC
