# Supplementary material for: Delta‐like ligand‐4 regulates Notch‐mediated maturation of second heart field progenitor‐derived pharyngeal arterial endothelial cells
Source: J Cell Mol Med. 2022 Sep 9;26(20):5181–94. doi: 10.1111/jcmm.17542 (PMC9575135; doi:10.1111/jcmm.17542)
Supplement: Supplementary file 4 — Table S2 [file JCMM-26-5181-s001.pdf]

Table S2

Antibody, Vector Information, Cell Lines and Chemicals		
**Antibody**	**Catalog number**	**Working Concentration /Dilution**
**Dll4** (Rabbit polyclonal)	Ab7280 (Abcam)	20µg/mL
**Dll4** (Rabbit polyclonal)	PA1-86891 (Thermofisher)	5µg/mL
**Islet1** (Goat Polyclonal)	AF1837 (R&D)	10µg/mL
**pHH3** (Rabbit polyclonal)	9701S (Cell Signaling)	0.1µg/mL
**CD31** (Goat Polyclonal)	AF3628 (R&D)	1µg/mL
**CD31** (Rat Monoclonal)	550274 (BD Pharmingen)	1:100
**EphrinB2** (Goat Polyclonal)	AF496 (R&D)	1µg/mL
**EphB4** (Goat Polyclonal)	AF446 (R&D)	1µg/mL
**Neuropilin1** (Goat Polyclonal)	AF566 (R&D)	10µg/mL
**Neuropilin2** (Goat Polyclonal)	AF567 (R&D)	10µg/mL
**Hey1** (Rabbit Polyclonal)	NBP2-16818 (Novus)	1:100
**SMα** (Rabbit Polyclonal)	NBP1-30894 (Novus)	1:100
**Notch1** (Rabbit Monoclonal)	EP1238Y (Abcam)	1:100
**Notch4** (Rabbit Monoclonal)	EPR18049 (Abcam)	1:200
**Jagged1** (Rabbit Polyclonal)	PA5-86057 (Invitrogen)	1:50
**Anti-Digoxigenin-POD, Fab fragments**	11207733910 (Roche)	0.5U/mL
**VEGF 165 Protein**	293-VE-050/CF (R&D)	2.5ng/mL
**Cell lines**		
**293T/17 [HEK 293T/17]**	CRL-11268 (ATCC)	
**Human Umbilical Artery Endothelial Cells**	C12202 (PromoCell)	
